# E-cigarettes and flavorings induce inflammatory and pro-senescence responses in oral epithelial cells and periodontal fibroblasts

**DOI:** 10.18632/oncotarget.12857

**Published:** 2016-10-24

**Authors:** Isaac K. Sundar, Fawad Javed, Georgios E. Romanos, Irfan Rahman

**Affiliations:** ^1^ Department of Environmental Medicine, University of Rochester Medical Center, Rochester, NY; ^2^ Department of General Dentistry, Eastman Institute for Oral Health University of Rochester, Rochester, NY; ^3^ Department of Periodontology, School of Dental Medicine, Stony Brook University, Stony Brook, NY

**Keywords:** e-cigarettes, e-juices, RAGE, COX_2_, PGE_2_

## Abstract

Electronic-cigarettes (e-cigs) represent a significant and increasing proportion of tobacco product consumption, which may pose an oral health concern. Oxidative/carbonyl stress via protein carbonylation is an important factor in causing inflammation and DNA damage. This results in stress-induced premature senescence (a state of irreversible growth arrest which re-enforces chronic inflammation) in gingival epithelium, which may contribute to the pathogenesis of oral diseases. We show that e-cigs with flavorings cause increased oxidative/carbonyl stress and inflammatory cytokine release in human periodontal ligament fibroblasts, Human Gingival Epithelium Progenitors pooled (HGEPp), and epigingival 3D epithelium. We further show increased levels of prostaglandin-E2 and cycloxygenase-2 are associated with upregulation of the receptor for advanced glycation end products (RAGE) by e-cig exposure-mediated carbonyl stress in gingival epithelium/tissue. Further, e-cigs cause increased oxidative/carbonyl and inflammatory responses, and DNA damage along with histone deacetylase 2 (HDAC2) reduction via RAGE-dependent mechanisms in gingival epithelium. A greater response is elicited by flavored e-cigs. Increased oxidative stress, pro-inflammatory and pro-senescence responses (DNA damage and HDAC2 reduction) can result in dysregulated repair due to proinflammatory and pro-senescence responses in periodontal cells. These data highlight the pathologic role of e-cig aerosol and its flavoring to cells and tissues of the oral cavity in compromised oral health.

## INTRODUCTION

The use of electronic-cigarettes (e-cigs) is increasing in the United States, which may pose oral health concerns [1]. E-cigs are battery operated devices, which consist of a metal heating element in a stainless steel shell, a cartridge, an atomizer and a battery. The heating element vaporizes a solution containing a mixture of chemicals including nicotine and other additives/humectants, such as base/carrying agents propylene glycol, glycerin/glycerol, and flavoring agents including fruit and candy flavors. Apart from inhaled nicotine, variable levels of aldehydes and carbonyls are detected in e-cig aerosols during vaporizations [2, 3]. Aldehyde causes carbonyl/oxidative stress, DNA adducts/damage, as well as stress-induced cellular senescence (a state of irreversible growth arrest which re-enforces chronic inflammation) [4, 5] leading to oral health problems [6–8].

Periodontal disease is characterized by chronic inflammation of the supporting tissues of the teeth. Periodontal ligament and gingival fibroblasts as well as epithelial cells are the most abundant structural cells in periodontal tissue. Upon stimulation or stress, these cells are able to incite and maintain inflammatory responses [9]. There is an association between smoking and tooth loss, periodontal attachment level, deeper periodontal pockets, and more extensive alveolar bone loss along with the destruction of connective tissue and matrix, leading to increased risk of periodontitis [10]. Clinical studies [11, 12] have also shown that habitual tobacco smokers exhibit a greater number of sites with plaque accumulation, clinical attachment loss and probing depth (≥ 4 mm) as compared to individuals who had never used tobacco in any form. It is important to mention that bleeding upon probing (a classical marker of periodontal disease activity) is masked in tobacco smokers than non-smokers [11, 12]. This most likely occurs as a result of the vasoconstrictive effect of nicotine (nicotine is the main component in e-cigs) on gingival blood vessels. Therefore, tobacco smokers may remain unaware of ongoing periodontal destruction until the inflammatory process reaches a stage where tooth mobility becomes evident.

We have recently shown the comparable oxidants/reactive oxygen species (ROS) reactivity in e-cig aerosols compared to conventional cigarette smoke [13, 14]. Smoking tobacco contributes to the progression of periodontal disease [10]. However, there is no information available regarding the e-cig aerosols vaping on periodontal/gingival oral health effects, especially in response to e-cig flavoring agents and nicotine. Periodontal/gingival cells in the oral cavity are the first targets by aerosols of e-cigs. Furthermore, the effects of e-cig aerosols on carbonyl stress, inflammation, and pro-senescence have not been studied on oral health. Here, we have determined the mechanism of gingival epithelial inflammation and pro-senescence by e-cig aerosols with flavorings in human oral epithelial cells and periodontal ligament fibroblasts.

## RESULTS

### E-cigarette exposure in human periodontal ligament fibroblasts (HPdLFs) and human gingival epithelium progenitors, pooled (HGEPp) increases protein carbonylation and pro-inflammatory responses

**Figure 1:**
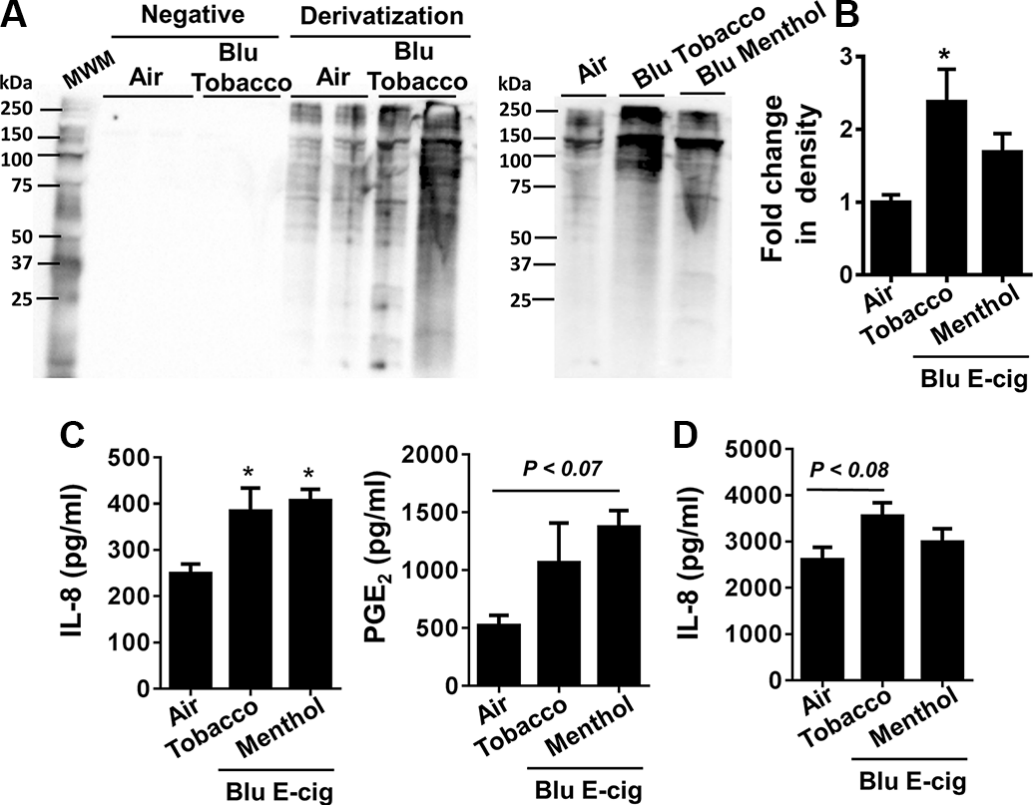
E-cig aerosol resulted in increased protein carbonylation and inflammatory responses in human periodontal ligament fibroblasts (HPdLFs) and human gingival epithelium progenitors, pooled (HGEPp) HPdLFs (**A**–**C**) and HGEPp (**D**) were exposed to aerosols from BLU® e-cig (Classic Tobacco, 16 mg nicotine; and Magnificent Menthol, ‘zero’ nicotine) (2 puffs/min; 4–5 sec/puff every 25 sec) using air-liquid interface system for 15 min, and then incubated at 37°C and 5% CO2 for 24 h. (A, B) Protein carbonylation in cell lysates was determined by the oxyblots. Gel pictures shown are representative of at least 3 separate experiments for protein carbonylation. (C, D) Levels of IL-8 and PGE2 in supernatants were determined by ELISA. Data are means ± SE (*n* = 3–6/group) and significance determined using 1-way ANOVA. *P < 0.05, vs. air. MWM: Molecular weight markers; Negative: without derivitizing reagent 2,4-dinitrophenylhydrazine (DNPH).

It is possible that ROS produced by e-cig vapors in cultured cells can augment protein carbonylation. The oxyblot assay was performed for immunodetection of the carbonyl groups that were introduced into protein side chains by e-cig-induced oxidative stress. We found that e-cig flavoring (BLU^®^ Classic Tobacco, 16 mg nicotine and Magnificent Menthol, zero nicotine) had different levels of protein oxidation as confirmed by OxyBlot. E-cigarette vapors from BLU^®^ Classic Tobacco showed significant increase in protein carbonylation compared to air exposed controls. E-cigarette vapors from BLU^®^ Magnificent Menthol flavor also showed a trend towards increased protein carbonylation but not significant compared to the control (Figure 1A–1B).

HPdLFs and HGEPp were exposed to BLU^®^ e-cig vapors (Classic Tobacco, 16 mg nicotine; and Magnificent Menthol, zero nicotine) using ALI system for 15 min. Conditioned medium was collected from both air exposed (control) and e-cig vapor exposed cells 24 hrs post ALI exposure. IL-8 secretion in conditioned medium 24 hrs after ALI exposure was significantly increased in both BLU^®^ e-cig vapors. PGE_2_ secretion showed a trend of incremental increase, but was not significant compared to air exposed controls (Figure 1C). IL-8 release was significantly higher in both BLU^®^ Classic Tobacco (16 mg nicotine) and Magnificent Menthol (zero nicotine) e-cig vapor exposed HPdLF cells compared to the controls. PGE_2_release was significantly higher in BLU^®^ Magnificent Menthol (zero nicotine) e-cig vapor exposed cells compared to the controls (Figure 1C). Similarly, HGEPp cells also showed an increased trend in IL-8 release in BLU^®^ Classic Tobacco (16 mg nicotine) e-cig vapor exposed cells compared to the controls. We did not see a significant increase in IL-8 release in HGEPp cells exposed to BLU^®^ Magnificent Menthol (zero nicotine) e-cig vapor compared to control (Figure 1D). Overall, e-cig vapors with flavoring induce protein carbonylation and increase in pro-inflammatory cytokines release which are indicative of oxidative stress and inflammatory response cause by e-cig vapors in HPdLF and HGEPp.

### E-cigarette exposure in human periodontal ligament fibroblasts (HPdLFs) increases inflammation and DNA damage markers

**Figure 2:**
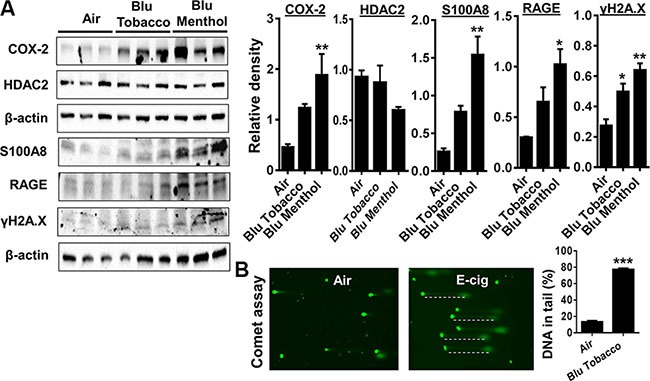
E-cig vapor exposure caused inflammatory responses and DNA damage in human periodontal ligament fibroblasts (HPdLFs) (**A**) HPdLFs were exposed to aerosols from BLU® e-cig (Classic Tobacco, 16 mg nicotine; and Magnificent Menthol, ‘zero’ nicotine) for 15 min (2 puffs/min; 4–5 sec/puff every 25 sec) using air-liquid interface system, and then incubated at 37°C and 5% CO2 for 24 h. (A) Levels of COX-2, S100A8, RAGE, γH2AX, and HDAC2 in cell lysates were measured by Western blotting. (**B**) DNA damage, indicated by an increase in fluorescent tail length, was measured by the Comet assay. Dash lines indicate DNA in tails. Data are means ± SE (*n* = 3–6/group) and significance determined using 1-way ANOVA or 2-tail *t*-test. *P < 0.05, **P < 0.01, ***P < 0.001

HPdLFs exposed to e-cig vapor from BLU^®^ e-cig (Classic Tobacco, 16 mg nicotine; and Magnificent Menthol, zero nicotine) for 15 min using ALI system and incubated for 24 h. Then, we measured several markers of inflammation such as PGE_2_-mediated COX-2 induction, HDAC2, S100A8, RAGE and phosphorylated γH2A.X (Ser139) in whole cell lysates. We found E-cig flavoring BLU^®^ Classic Tobacco (16 mg nicotine) and Magnificent Menthol (zero nicotine) showed a differential effect on levels of COX-2, HDAC2, S100A8, RAGE and γH2A.X in HPdLFs *in vitro* (Figure 2A). HPdLFs exposed to BLU^®^ e-cig vapors (Magnificent Menthol, zero nicotine) showed a significant increase in all the inflammatory markers (COX-2, S100A8, RAGE), a trend towards reduced HDAC2 and increased phosphorylated γH2A.X (Ser139), a DNA damage marker compared to control. HPdLFs exposed to BLU^®^ e-cig (Classic Tobacco, 16 mg nicotine) also showed a significant increase in S100A8 and γH2A.X, and an increased trend in COX-2 compared to the controls. We did not observe any significant increase in RAGE or a significant decrease in HDAC2 levels in BLU^®^ E-cig vapor (Classic Tobacco, 16 mg nicotine) exposed HPdLFs compared to the controls (Figure 2A). Likewise, the HPdLFs exposed to e-cig vapor from BLU^®^ e-cig (Classic Tobacco, 16 mg nicotine) showed a significant increase in DNA damage as measured by the percentage of DNA in tail using the Comet assay (Figure 2B). Overall, we showed that e-cig vapors with flavoring differentially affects inflammatory response and DNA damage markers as a result of oxidative stress and inflammatory response caused by e-cig vapors in HPdLFs.

### E-cigarette exposure in human gingival epithelium progenitors, pooled (HGEPp) increases inflammation and DNA damage markers

**Figure 3:**
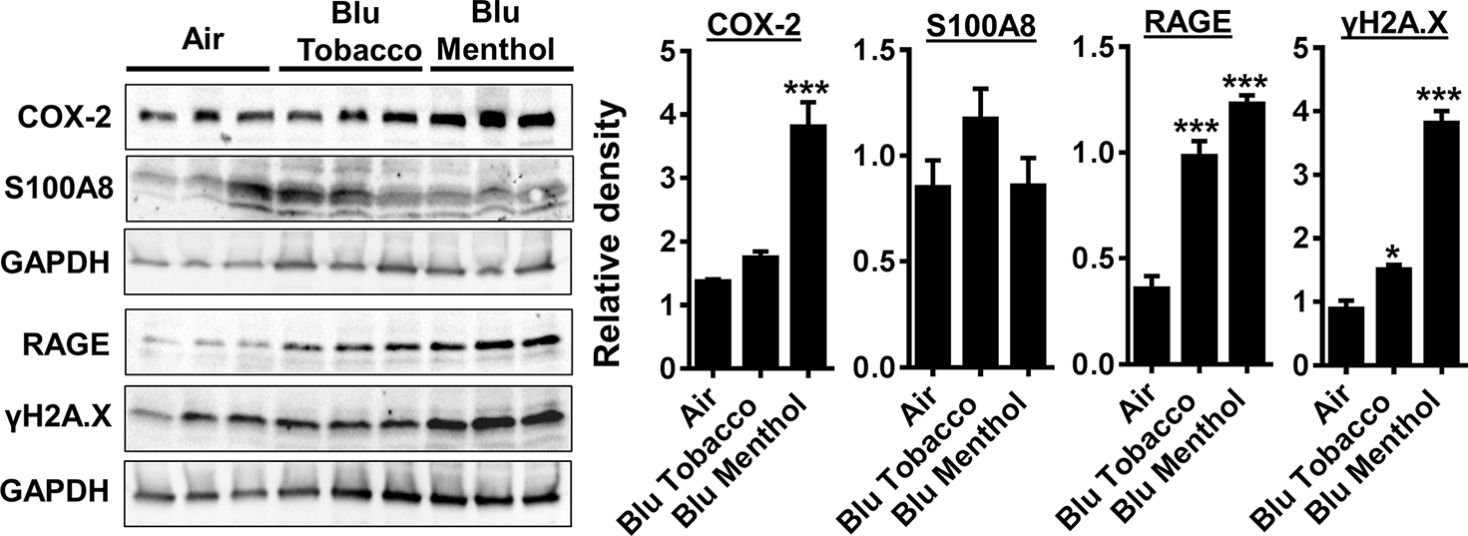
E-cig vapor exposure caused inflammatory responses and DNA damage in human gingival epithelium progenitors, pooled cells (HGEPp) (**A**) HGEPp cells were exposed to aerosols from BLU^®^ e-cig (Classic Tobacco, and Magnificent Menthol) (2 puffs/min; 4–5 sec/puff every 25 sec) using air-liquid interface system for 15 min, and then incubated at 37°C and 5% CO_2_ for 24 h. Levels of COX-2, S100A8, RAGE, and γH2AX in cell lysates were measured by Western blotting. Data are means ± SE (*n* = 3–6/group) and significance determined using 1-way ANOVA. *P < 0.05, ***P < 0.001, vs. air.

We found E-cig flavoring BLU^®^ Classic Tobacco (16 mg nicotine) and Magnificent Menthol (zero nicotine) showed differential effects on levels of COX-2, S100A8, RAGE and γH2A.X in HGEPp cells *in vitro* (Figure 2A). HGEPp cells exposed to BLU^®^ e-cig vapors (Classic Tobacco, 16 mg nicotine and Magnificent Menthol, zero nicotine) showed a significant increase in the inflammatory markers (COX-2 and RAGE), and DNA damage marker (phosphorylated γH2A.X Ser139) compared to the controls. The effect of BLU^®^ e-cig vapors (Magnificent Menthol, zero nicotine) on HGEPp cells was significantly higher compared to BLU^®^ e-cig vapors Classic Tobacco (16 mg nicotine) and the controls. We did not observe any significant increase in COX-2 and S100A8 levels in BLU^®^ e-cig vapor from Classic Tobacco (16 mg nicotine) as well as S100A8 levels in Magnificent Menthol (zero nicotine) exposed HGEPp cells compared to the controls (Figure 3). Overall, we confirmed that e-cig vapors with flavoring affects inflammatory response and DNA damage markers as a result of oxidative stress and inflammatory response caused by e-cig vapors in HGEPp cells.

### E-cigarette exposure in human 3D *in vitro* model of EpiGingival tissue increases inflammation and DNA damage markers

**Figure 4:**
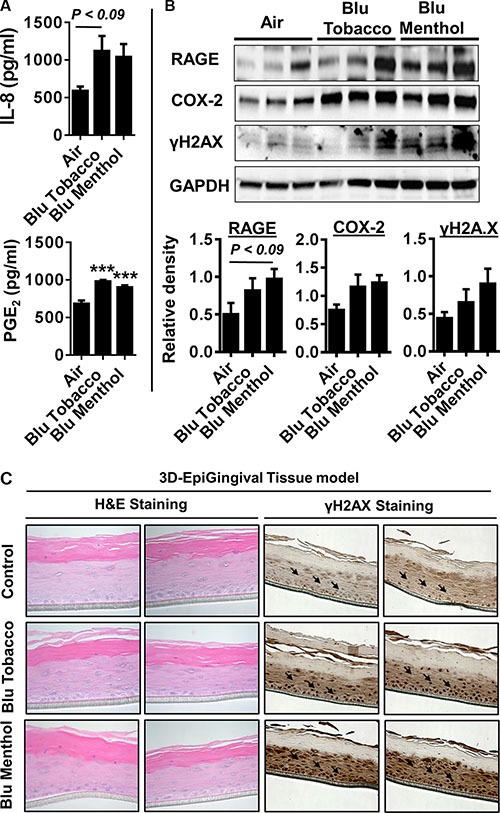
E-cig vapor caused inflammatory responses and DNA damage in normal human 3D *in vitro* model of EpiGingival tissues Normal human 3D *in vitro* model of EpiGingival tissue (Cat#: GIN-100, MatTek) were exposed to aerosols from BLU^®^ e-cig (Classic Tobacco, and Magnificent Menthol) using air-liquid interface system for 15 min, and then incubated at 37°C and 5% CO_2_ for 24 h. (**A**) Levels of IL-8 and PGE_2_ in culture media were determined by ELISA. (**B**) Levels of RAGE, COX-2, and γH2AX in tissue lysates were measured by Western blotting. (**C**) Representative images of EpiGingival tissues used for ALI exposures stained with H&E (showing histological features) and γH2AX staining 24 h post exposure to control (air) and flavored e-cig aerosols. Immunohistochemistry revealed a distinct staining in both the flavored BLU e-cig exposed EpiGingival tissues for γH2AX. Data are means ± SE (*n* = 4–6/group) and significance determined using 1-way ANOVA. ***P < 0.001,vs. air.

Human 3D model of EpiGingival tissues were exposed to e-cig vapor from BLU^®^ (Classic Tobacco, 16 mg nicotine; and Magnificent Menthol, zero nicotine) for 15 min using the modified ALI system without culture media. The human 3D EpiGingival tissue insert was contained within 35 mm culture dishes along with 900 μl culture medium during e-cig vapor exposure, and incubated for 24 h. Then, we measured pro-inflammatory cytokines IL-8 and PGE_2_ in conditioned medium collected 24 h post last exposure. Markers of inflammation and DNA damage, such as RAGE, PGE_2_-mediated COX-2 induction and γH2A.X were measured using whole tissue lysates from 3D EpiGingival cultures. We found e-cig flavoring BLU^®^Classic Tobacco (16 mg nicotine) and Magnificent Menthol (zero nicotine) showed an increasing trend in the levels of IL-8 release and a significant increase in the levels of PGE_2_ release compared to the controls (Figure 4A). Additionally, only e-cig flavoring BLU^®^ Magnificent Menthol (zero nicotine) showed an increasing trend in RAGE, COX-2, and γH2A.X (immunoblot analysis and immunohistochemistry) in 3D EpiGingival tissues *in vitro* compared to the controls (Figure 4B–4C). The effect of BLU^®^ e-cig vapors (Magnificent Menthol, zero nicotine) on 3D EpiGingival tissues was significantly higher compared to BLU^®^ e-cig vapors Classic Tobacco (16 mg nicotine) and the controls. Overall, we reproduced the above findings in 3D models that e-cig vapors with flavoring differentially affects inflammatory response and DNA damage markers as a result of oxidative stress and inflammatory response caused by e-cig vapors in 3D *in vitro* model of EpiGingival tissues.

## DISCUSSION

In this study, we show that e-cigs with flavorings cause increased oxidative/carbonyl stress and inflammatory cytokine release in human periodontal ligament fibroblasts, Human Gingival Epithelium Progenitors pooled (HGEPp), and EpiGingival 3D epithelium. We further show increased levels of prostaglandin-E_2_ and cycloxygenase-2 were associated with upregulation of the receptor for advanced glycation end products (RAGE) by e-cig exposure-mediated carbonyl stress in gingival epithelium/tissue. Further, e-cigs cause increased oxidative/carbonyl stress and inflammatory responses, and DNA damage along with histone deacetylase 2 (HDAC2) reduction via RAGE-dependent mechanisms in gingival epithelium, with greater response by flavored e-cigs. Increased oxidative stress, pro-inflammatory and pro-senescence responses (DNA damage and HDAC2 reduction) can result in dysregulated repair due to proinflammatory and pro-senescence responses in periodontal cells. Our data also implicate that e-cig affects the regenerative potential of human progenitor cells due to increased inflammatory and DNA damage responses. It is well known that the mTOR pathway is activated by most oncogenes that induce cellular senescence (i.e., by Ras, Raf, MEK, and Akt). Further, the mechanistic target of rapamycin (mTOR) is generally activated in proliferating cells. During acute DNA damage, mTOR induces DNA damage response (DDR) and cell cycle arrest (induction of p21 and 16) [15]. Such arrested cells where mTOR is active play an important role in geroconversion (converts quiescence into senescence) to their pro-senescent phenotype [15, 16]. In case of oncogene-induced senescence, DDR causes cell cycle arrest, leading to cell senescence [16]. This may be one of the mechanisms of e-cigarette induce DDR response via mTOR activation.

Conventional cigarette smoke has been shown to cause deleterious effects on oral and periodontal health [10]. However, the role of e-cig vaping and its association with carbonyl stress, inflammation, and DNA damage-triggered senescence on oral/periodontal epithelium remains unknown. Periodontal ligament and gingival fibroblasts as well as epithelial cells are the most abundant structural cells in periodontal tissue, and are the direct targets of e-cigs upon vaping. Upon stimulation or stress, these cells are able to incite and maintain inflammatory responses [9]. There is an association between smoking and tooth loss, periodontal attachment loss, deeper periodontal pockets, and more extensive alveolar bone loss along with the destruction of connective tissue and matrix, leading to an increased risk of periodontitis [10]. However, no studies are available to document the effects of e-cig vaping especially in response to e-cig flavoring agents on periodontal health in terms of oxidative/carbonyl stress and inflammation in human gingival epithelial cells *in vitro*.

We have recently shown oxidants/ROS reactivity from e-cig aerosols is comparable to conventional cigarette smoke [13, 14]. We show that e-cig flavoring caused protein oxidation as reflected in increased protein carbonylation. This was associated with increased IL-8 and PGE_2_ secretion from HPdLFs and HGEPp cells upon exposure to e-cig aerosols. Direct exposure to e-liquids (this is not the case when users vape e-cigs i.e. users do not consume e-liquids) has also been shown to produce harmful effects in periodontal ligament and gingival fibroblasts in culture [17, 18]. While the contribution of smoking tobacco to the progression of periodontal disease and other adverse oral health outcomes is well described [10, 19], no information is available regarding the impact of e-cig aerosols vaping on periodontal/gingival oral health effects, especially in response to e-cig flavoring agents. We determined the effect of flavoring on oxidative and pro-inflammatory responses, and upon exposure of periodontal ligament fibroblasts, Human Gingival Epithelium Progenitors pooled (HGEPp), and epigingival 3D epithelium to menthol flavoring agent resulted in increased oxidative/carbonyl stress, and IL-8 release. Menthol acts on transient receptor potential ankyrin 1 (TRPA1) receptors to activate inflammatory responses [20, 21]. It may be conceived that activation of TRPA receptors by e-cig aerosols will drive COX-PGE_2_ mediated responses in periodontal tissues, leading to augmentation of inflammatory and pro-fibrotic and pro-carcinogenic responses. However, further studies are required to understand the augmented response by BLU^®^ menthol flavoring than BLU^®^ classic tobacco.

Protein carbonylation leads to autoantibody production which may lead to destruction of matrix and bone loss during periodontitis [6, 7]. Further, it is possible that carbonyls/aldehydes play an important role in e-cig aerosol-induced oral toxicity. Conventional tobacco smoke is known to cause oxidative burden leading to DNA damage and inflammatory responses [22]. The RAGE is a pattern-recognition receptor implicated in immune and inflammatory diseases including dental pulp inflammation and periodontitis [23–27]. RAGE is involved in smoking-related disorders and known to cause cellular senescence via oxidant stress [28, 29]. However, the mechanism of RAGE-mediated signaling especially via its ligand S100A8 as a susceptible factor in inducing gingival epithelial inflammation and senescence by e-cigs is not known. E-cig vapor exhibited significant inflammatory response (COX2, RAGE S100A8), DNA damage as determined by the Comet assay and γ-H2AX levels (a marker of DNA damage) in gingival epithelium/tissue. Our data showing inflammatory and pro-DNA damage responses are unique in light of primary cells and 3D tissue culture models which mimic closely with users vaping of e-cigs. Our results further attest the pro-oxidant, DNA damaging and pro-inflammatory effects of e-cig vapor exposure. The increased levels of pro-inflammatory mediators IL-8 and PGE_2_ would cause remodeling of the ECM during periodontitis by e-cig due to cellular senescence, where these cells have secretory phenotype to perpetuate the inflammatory responses.

It has been reported that outcomes of periodontal therapy are compromised in smokers compared with non-smokers [30, 31]. A variety of mechanisms have been proposed in this regard. For example, increased expression of RAGE occurs in gingival epithelial cells of smokers as compared to non-smokers. Furthermore, it has been reported that nornicotine (a metabolite of nicotine), upregulates RAGE expression in the gingivae of smokers and elicits a proinflammatory response by stimulating the secretion of cytokines and ROS which are involved in destruction of the periodontal tissue [32]. The vasoconstrictive effects of nicotine increase platelet adhesiveness, increases the risk of microvascular occlusion and causes tissue ischemia [32]. Furthermore, tobacco smoking is also associated with catecholamines release resulting in vasoconstriction and decreased tissue perfusion [33]. Therefore, it is hypothesized that the outcomes of periodontal surgery are compromised in e-cig users compared with non-smokers/non-users through the mechanisms comparable to those stated above. However, further studies are needed to test this contention in a clinical cohort of users and non-users of e-cigs vs conventional smokers.

In conclusion, our data showed that e-cig aerosol cause increased oxidative/carbonyl stress and inflammatory responses, and cellular senescence associated with persistent DNA damage via RAGE-HDAC2-dependent mechanisms in gingival epithelium, with greater response by flavored e-cigs. Further understanding of the chronic effect of vaping could lead to molecular mechanisms for susceptibility (inflammatory, DNA damage and senescence responses) to the development of periodontitis, and therapeutic targets or biomarkers in determining vaping-flavoring mediated oral complications in cells and tissues of the oral cavity. Our data also implicate that e-cig affects the regenerative potential of human progenitor cells due to increased inflammatory and DNA damage responses. Overall, our data suggest the pathogenic role of e-cig aerosol to cells and tissues of the oral cavity, leading to compromised periodontal health.

## MATERIALS AND METHODS

### E-cigarettes

BLU^®^ rechargeable e-cigs (Lorillard Technologies, Inc.) containing two different disposable cartomizers [Flavors: classic tobacco (16 mg nicotine), and magnificent menthol (zero nicotine or 13–16 mg nicotine)] with pre-loaded e-liquid were used. The BLU® e-cigarette device and disposable cartomizer cartridges were purchased from local retailers.

### Cell culture and air-liquid interface (ALI) culture/exposures

Clonetics™ Human periodontal ligament fibroblast (HPdLF, CC-7049; Lonza) were grown at 37°C in 5% CO_2_ incubator to 80–90% confluence in stromal cell medium (SCGM™ BulletKit, Lonza; CC-3205) supplemented with hFGF-B (0.5 ml), insulin (0.5 ml), FBS (25 ml) and GA-1000 (0.5 ml) according to manufacturer recommendations. Human gingival epithelium progenitors, (HGEPp; gingival epithelial cells) were grown in CnT-Prime medium (CnT-PR), epithelial culture medium as recommended by CELLnTEC Advanced Cell Systems. Transwell cultures were then placed into air-liquid interface (ALI) exposure chamber [14, 34]. BLU^®^ e-cigarette vapor (Classic Tobacco containing 16 mg nicotine or Magnificent Menthol flavor containing zero or 13–16 mg nicotine) was drawn into the ALI exposure chamber (2 puffs every 1 min), 4–5 second puff followed by 25 second pause for different time durations 5, 10, and 15 minutes respectively.

### Human EpiGingival tissue model

Human gingival tissues (EpiGingival, GIN-100) were obtained from MatTek Corporation (Ashland, MA). The 3D tissue model is a reconstructed oral epithelial tissue that are derived from human primary oral keratinocytes. This model and allowed to differentiate to a structure characteristic to that of *in vivo*. EpiGingival tissues were exposed to air (control) or BLU^®^ e-cigarette vapors (Classic Tobacco and Magnificent Menthol). After 15 min exposure, the conditioned medium was collected to measure pro-inflammatory cytokines and tissues harvested for Western blotting and immunohistochemistry according to the recommendations of the manufacturer. The protein levels were measured using a BCA kit (Pierce, IL, USA).

### Comet assay

Comet assay was performed as per the instructions of the manufacturer (Trevigen, Gaithersburg, MD) [34]. Comet images were captured using a Nikon ECLIPSE Ni fluorescent microscope. The images were analyzed using OpenComet software. The extent of DNA damage was expressed as a measure of percentage of DNA in tail.

### Protein carbonylation/oxyblot

Protein oxidation was determined using the OxyBlot protein oxidation detection kit following the manufacturer's instruction (Millipore, S7150). Equal amount of protein was loaded for oxyblot analysis, and the results were quantified by densitometry using Image J.

### Pro-inflammatory cytokine analysis

Following 24 hrs after BLU^®^ e-cigarette vapor exposure using air-liquid interface approach, conditioned media was collected and stored at −80°C for measuring pro-inflammatory mediators. IL-8 (Life Technologies, Carlsbad, CA) and PGE_2_ (Cayman Chemical, Ann Arbor, MI) levels were measured by enzyme-linked immunosorbent assay (ELISA) according to manufacturer's instructions.

### Western blotting

For Western blots, 25 μg protein samples were separated on a 4–15% gradient and 7.5% SDS-PAGE gels. Then probed with specific primary antibodies (1:1000 dilution in 5% milk in PBS containing 0.1% Tween 20 (v/v), such as anti-COX2 (Cayman chemical; #160112), HDAC2 (ab32117), S100A8 (ab92331), RAGE (ab37647) and γH2A.X phospho S139 (ab2893) from Abcam at 4°C overnight. The bound complexes were detected using ECL method with the ChemiDoc™ MP Imaging System (Bio-Rad). Equal loading of the samples was determined by quantitation of proteins and by stripping and re-probing membranes for β-actin or GAPDH (Santa Cruz Biotechnology, sc-1616 and sc-365062) and the results were quantified by densitometry using Image J.

### Statistical analysis

Statistical analysis of significance was calculated using unpaired Student's *t-test* for comparison between two groups control vs. e-cigarette. Probability of significance compared to control for more than two treatment groups (different e-cigarette flavors) was analyzed by 1-way ANOVA (Tukey's multiple comparisons test) using GraphPad Prism 6 as indicated in figure legends. The results are shown as the mean ± SEM unless otherwise indicated. *P* < 0.05 is considered as statistically significant.

## References

[1] Regan AK, Promoff G, Dube SR, Arrazola R (2013). Electronic nicotine delivery systems: adult use and awareness of the ‘e-cigarette’ in the USA. Tobacco control.

[2] Cheng T (2014). Chemical evaluation of electronic cigarettes. Tobacco control.

[3] Kosmider L, Sobczak A, Fik M, Knysak J, Zaciera M, Kurek J, Goniewicz ML (2014). Carbonyl compounds in electronic cigarette vapors: effects of nicotine solvent and battery output voltage. Nicotine & tobacco research : official journal of the Society for Research on Nicotine and Tobacco.

[4] Baraibar MA, Liu L, Ahmed EK, Friguet B (2012). Protein oxidative damage at the crossroads of cellular senescence, aging, and age-related diseases. Oxidative medicine and cellular longevity.

[5] Satoh R, Kishino K, Morshed SR, Takayama F, Otsuki S, Suzuki F, Hashimoto K, Kikuchi H, Nishikawa H, Yasui T, Sakagami H (2005). Changes in fluoride sensitivity during *in vitro* senescence of normal human oral cells. Anticancer research.

[6] Pradeep AR, Ramchandraprasad MV, Bajaj P, Rao NS, Agarwal E (2013). Protein carbonyl: An oxidative stress marker in gingival crevicular fluid in healthy, gingivitis, and chronic periodontitis subjects. Contemporary clinical dentistry.

[7] Baltacioglu E, Akalin FA, Alver A, Deger O, Karabulut E (2008). Protein carbonyl levels in serum and gingival crevicular fluid in patients with chronic periodontitis. Archives of oral biology.

[8] Canakci CF, Tatar A, Canakci V, Cicek Y, Oztas S, Orbak R (2006). New evidence of premature oxidative DNA damage: mitochondrial DNA deletion in gingival tissue of patients with periodontitis. Journal of periodontology.

[9] Ara T, Kurata K, Hirai K, Uchihashi T, Uematsu T, Imamura Y, Furusawa K, Kurihara S, Wang PL (2009). Human gingival fibroblasts are critical in sustaining inflammation in periodontal disease. Journal of periodontal research.

[10] Javed F, Bashir Ahmed H, Romanos GE (2014). Association between environmental tobacco smoke and periodontal disease: a systematic review. Environmental research.

[11] Javed F, Al-Askar M, Samaranayake LP, Al-Hezaimi K (2013). Periodontal disease in habitual cigarette smokers and nonsmokers with and without prediabetes. The American journal of the medical sciences.

[12] Javed F, Nasstrom K, Benchimol D, Altamash M, Klinge B, Engstrom PE (2007). Comparison of periodontal and socioeconomic status between subjects with type 2 diabetes mellitus and non-diabetic controls. Journal of periodontology.

[13] Lerner CA, Sundar IK, Watson RM, Elder A, Jones R, Done D, Kurtzman R, Ossip DJ, Robinson R, McIntosh S, Rahman I (2015). Environmental health hazards of e-cigarettes and their components: Oxidants and copper in e-cigarette aerosols. Environmental pollution.

[14] Lerner CA, Rutagarama P, Ahmad T, Sundar IK, Elder A, Rahman I (2016). Electronic cigarette aerosols and copper nanoparticles induce mitochondrial stress and promote DNA fragmentation in lung fibroblasts. Biochemical and biophysical research communications.

[15] Blagosklonny MV (2012). Cell cycle arrest is not yet senescence, which is not just cell cycle arrest: terminology for TOR-driven aging. Aging (Albany NY).

[16] Blagosklonny MV (2011). Molecular damage in cancer: an argument for mTOR-driven aging. Aging (Albany NY).

[17] Sancilio S, Gallorini M, Cataldi A, di Giacomo V (2016). Cytotoxicity and apoptosis induction by e-cigarette fluids in human gingival fibroblasts. Clinical oral investigations.

[18] Willershausen I, Wolf T, Weyer V, Sader R, Ghanaati S, Willershausen B (2014). Influence of E-smoking liquids on human periodontal ligament fibroblasts. Head & face medicine.

[19] Dodani K, Anumala N, Avula H, Reddy K, Varre S, Kalakonda BB, Arora N, Suri C, Avula JK (2012). Periodontal findings in patients with oral submucous fibrosis and comet assay of affected gingival epithelial cells. Journal of periodontology.

[20] Willis DN, Liu B, Ha MA, Jordt SE, Morris JB (2011). Menthol attenuates respiratory irritation responses to multiple cigarette smoke irritants. FASEB journal : official publication of the Federation of American Societies for Experimental Biology.

[21] Ha MA, Smith GJ, Cichocki JA, Fan L, Liu YS, Caceres AI, Jordt SE, Morris JB (2015). Menthol attenuates respiratory irritation and elevates blood cotinine in cigarette smoke exposed mice. PloS one.

[22] Kode A, Yang SR, Rahman I (2006). Differential effects of cigarette smoke on oxidative stress and proinflammatory cytokine release in primary human airway epithelial cells and in a variety of transformed alveolar epithelial cells. Respiratory research.

[23] Tancharoen S, Tengrungsun T, Suddhasthira T, Kikuchi K, Vechvongvan N, Tokuda M, Maruyama I (2014). Overexpression of receptor for advanced glycation end products and high-mobility group box 1 in human dental pulp inflammation. Mediators of inflammation.

[24] Lalla E, Lamster IB, Schmidt AM (1998). Enhanced interaction of advanced glycation end products with their cellular receptor RAGE: implications for the pathogenesis of accelerated periodontal disease in diabetes. Annals of periodontology / the American Academy of Periodontology.

[25] Lalla E, Lamster IB, Feit M, Huang L, Spessot A, Qu W, Kislinger T, Lu Y, Stern DM, Schmidt AM (2000). Blockade of RAGE suppresses periodontitis-associated bone loss in diabetic mice. The Journal of clinical investigation.

[26] Ito Y, Bhawal UK, Sasahira T, Toyama T, Sato T, Matsuda D, Nishikiori H, Kobayashi M, Sugiyama M, Hamada N, Arakawa H, Kuniyasu H (2012). Involvement of HMGB1 and RAGE in IL-1beta-induced gingival inflammation. Archives of oral biology.

[27] Xie J, Mendez JD, Mendez-Valenzuela V, Aguilar-Hernandez MM (2013). Cellular signalling of the receptor for advanced glycation end products (RAGE). Cellular signalling.

[28] Liu J, Huang K, Cai GY, Chen XM, Yang JR, Lin LR, Yang J, Huo BG, Zhan J, He YN (2014). Receptor for advanced glycation end-products promotes premature senescence of proximal tubular epithelial cells via activation of endoplasmic reticulum stress-dependent p21 signaling. Cellular signalling.

[29] Fang M, Wang J, Li S, Guo Y (2015). Advanced glycation end-products accelerate the cardiac aging process through the receptor for advanced glycation end-products/transforming growth factor-beta-Smad signaling pathway in cardiac fibroblasts. Geriatrics & gerontology international.

[30] Kotsakis GA, Javed F, Hinrichs JE, Karoussis IK, Romanos GE (2015). Impact of cigarette smoking on clinical outcomes of periodontal flap surgical procedures: a systematic review and meta-analysis. Journal of periodontology.

[31] Javed F, Al-Rasheed A, Almas K, Romanos GE, Al-Hezaimi K (2012). Effect of cigarette smoking on the clinical outcomes of periodontal surgical procedures. The American journal of the medical sciences.

[32] Katz J, Caudle RM, Bhattacharyya I, Stewart CM, Cohen DM (2005). Receptor for advanced glycation end product (RAGE) upregulation in human gingival fibroblasts incubated with nornicotine. Journal of periodontology.

[33] Balaji SM (2008). Tobacco smoking and surgical healing of oral tissues: a review. Indian journal of dental research : official publication of Indian Society for Dental Research.

[34] Lerner CA, Sundar IK, Yao H, Gerloff J, Ossip DJ, McIntosh S, Robinson R, Rahman I (2015). Vapors produced by electronic cigarettes and e-juices with flavorings induce toxicity, oxidative stress, and inflammatory response in lung epithelial cells and in mouse lung. PloS one.

